# fSpatial and temporal dynamics of cellulose degradation and biofilm formation by *Caldicellulosiruptor obsidiansis *and *Clostridium thermocellum*

**DOI:** 10.1186/2191-0855-1-30

**Published:** 2011-10-07

**Authors:** Zhi-Wu Wang, Seung-Hwan Lee, James G Elkins, Jennifer L Morrell-Falvey

**Affiliations:** 1 BioEnergy Science Center, Biosciences Division, Oak Ridge National Laboratory, Oak Ridge, TN 37831, USA; 2 National Institute of Advanced Industrial Science and Technology, Biomass Technology Research Center, Hiroshima, Japan

**Keywords:** biofilm, thermophile, cellulosome, cellulose

## Abstract

Cellulose degradation is one of the major bottlenecks of a consolidated bioprocess that employs cellulolytic bacterial cells as catalysts to produce biofuels from cellulosic biomass. In this study, we investigated the spatial and temporal dynamics of cellulose degradation by *Caldicellulosiruptfor obsidiansis*, which does not produce cellulosomes, and *Clostridium thermocellum*, which does produce cellulosomes. Results showed that the degradation of either regenerated or natural cellulose was synchronized with biofilm formation, a process characterized by the formation and fusion of numerous crater-like depressions on the cellulose surface. In addition, the dynamics of biofilm formation were similar in both bacteria, regardless of cellulosome production. Only the areas of cellulose surface colonized by microbes were significantly degraded, highlighting the essential role of the cellulolytic biofilm in cellulose utilization. After initial attachment, the microbial biofilm structure remained thin, uniform and dense throughout the experiment. A cellular automaton model, constructed under the assumption that the attached cells divide and produce daughter cells that contribute to the hydrolysis of the adjacent cellulose, can largely simulate the observed process of biofilm formation and cellulose degradation. This study presents a model, based on direct observation, correlating cellulolytic biofilm formation with cellulose degradation.

## Introduction

Biofuels provide a number of environmental advantages over fossil fuels, especially in greenhouse gas reduction (Hromadko et al. 2010). Cellulosic biomass is often recognized as one of the best resources for biofuel production based on its cost, abundance, and cleanliness (Lynd et al. 2008). The hydrolysis of cellulosic biomass into soluble sugar, however, is regarded as a rate-limiting step in cellulosic biofuel production (Lynd et al. 2002). Consolidated bioprocessing (CPB) which utilizes cellulolytic bacteria to directly convert biomass into biofuel has the potential to cost significantly less compared to methods using enzymes (Lynd et al. 2008). Despite numerous studies showing biofilm involvement in cellulosic biomass hydrolysis (Cheng et al. 1984; Mooney and Goodwin 1991; Weimer et al. 1993; Miron et al. 2001; Burrell et al. 2004; Song et al. 2005, Lynd et al. 2006), few details are known regarding the dynamic interaction between biofilm formation and cellulose degradation. Some cellulolytic bacteria, such as *Clostridium*, produce cellulosomes which are protein complexes that facilitate cell attachment to cellulose and provide docking sites for extracellular enzymes involved in biomass hydrolysis (Miron et al. 2001). Yet, not all cellulolytic bacteria produce cellulosomes and very little is known regarding the mechanisms by which these non-cellulosome producing microbes attach to and degrade cellulose (Lynd et al. 2006). *Caldicellulosiruptor obsidiansis *is an anaerobic non-cellulosome producing bacterium isolated from Yellowstone National Park with an optimal temperature for growth at 78°C (Hamilton-Brehm et al. 2009). This organism hydrolyzes both cellulose and hemicellulose while fermenting hexose and pentose sugars to produce hydrogen, organic acids and ethanol. In this study, the temporal and spatial interactions of *C. obsidiansis *with cellulose were visualized and compared to *C. thermocellum*. This study was undertaken with the goal of providing insights into the mechanisms of microbial cellulose utilization, especially in high temperature environments.

## Materials and methods

### Microbial growth

Commercially available regenerated cellulose membranes with 0.2 μm pore size (Whatman RC58, Maidstone, Kent, UK) or flat-surface cellulose membrane made of natural cotton linter nanofiber (Celish KY-100G, Daicel Chemical Industries, LTD, Osaka, Japan) were used as cellulose substrates in this study. The linter cellulose was microfibrillated by high-pressure homogenization and showed nanoscopic morphology, with a crystallinity index (Segal et al., 1959) of 82%.

Identical chads with a mean diameter of 7.37 ± 0.03 mm were stamped from both types of cellulose membrane and used as the sole carbon source to support the growth of *C. obsidiansis *(ATCC BAA2073) or *C. thermocellum *(ATCC27405) in liquid culture. Serum bottles, each containing one cellulose chad and 50 ml nutrient media, were inoculated with 2 × 10^5 ^ml^-1 ^cells and incubated under anaerobic conditions at 75°C for *C. obsidiansis *and 60°C for *C. thermocellum *with moderate shaking (100 rpm) and nitrogen gas headspace. Nutrient media for *C. obsidiansis *was prepared according to Hamilton-Brehm et al. (2009), with the exception that no yeast extract was added. Nutrient media for *C. thermocellum *was same as that used by Zhang and Lynd (2005). This experimental design gives an equivalent initial substrate concentration of 0.03 g cellulose L^-1^. Replicate serum bottles were prepared and 3 bottles were harvested every four hours for analysis.

### Microscopy

Sampled cellulose chads were stained with Syto9 (Invitrogen, Carlsbad, CA) to visualize the distribution of bacterial cells on the cellulose chad surface using confocal laser scanning microscopes (Leica TCS SP2, Mannheim, Germany or Zeiss LSM 710, Jena, Germany). The mean thickness of each regenerated cellulose chad was determined by measuring the change in the Z-dimension by focusing the confocal microscope on the top and bottom of the chad at 10 randomly chosen positions. The planktonic cell count was determined using a Thoma cell counting chamber (Blaubrand, Wertheim, Germany) and an Axioskop2 Plus microscope (Zeiss, Thornwood, NY, USA) with phase contrast illumination. ImageJ software (Version 1.42q, NIH, Bethesda, MD) was used for image analyais. The ImageJ 3D viewer plug-ins were installed to reconstruct the biofilm in three dimensions.

### Biofilm cell density determination

The cell density in the biofilm was determined using the object counter3D plug-in installed in ImageJ. Briefly, the software counts the number of objects scattered in a 3D space, which can be converted to cell density within the space volume. For this study, the number of objects within five randomly selected biofilm internal subspaces with dimension of 30 × 30 × 30 μm^3 ^were averaged to calculate cell density. For comparison with this study, the minimum cell density of biofilms reported in the literature was estimated using the following calculation. Because most published images show only monolayer biofilms, the cell density per area, namely ρ_a _(cells cm^-2^), was first calculated by counting the number of cells in a given area of the published image and converting this result to the minimum volumetric density, namely ρ_v _(cells cm^-3^). To do this, a maximum biofilm thickness can be estimated from the mean intercellular distance (d) calculated from,

(1) $$\text{d}=\frac{1}{\sqrt{\rho_{\text{a}}}}$$

And then, the minimum volumetric biofilm cells density can be approximated by,

(2) $$\rho_{\text{v}}=\frac{\rho_{\text{a}}}{\text{d}}$$

## Results

### Temporal and spatial dynamics of *C. obsidiansis *biofilm formation

To visualize the process of biofilm formation by *C. obsidiansis *on a model cellulose substrate, cells were grown in serum bottles containing a regenerated cellulose chad as the sole carbon source. Based on imaging data, the dynamic process of biofilm formation and growth can be differentiated into multiple steps, including: i) initial cell attachment to the substrate; ii) cell growth and division and iii) inverted colony formation; iv) crater-like depression formation due to degradation of the cellulose substrate by the microbial colony; v) fusion of the depressions due to continued growth and substrate degradation, leading to vi) a biofilm of uniform thickness.

### Initial microbial attachment and growth

**Figure 1:**
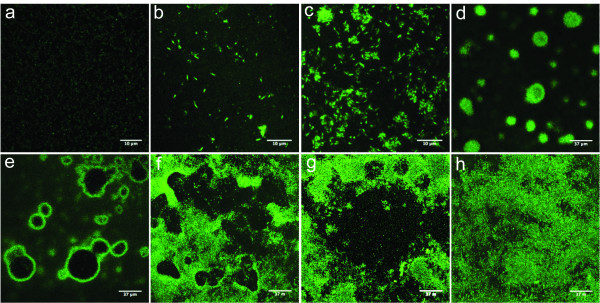
**Distribution of *C. obsidiansis *cells on a cellulose surface after incubation for a) 0 h, b) 8 h, c) 16 h, d) 24 h, e) 44 h, f) 48 h, g) 56 h and h) 68 h**.

**Figure 2:**
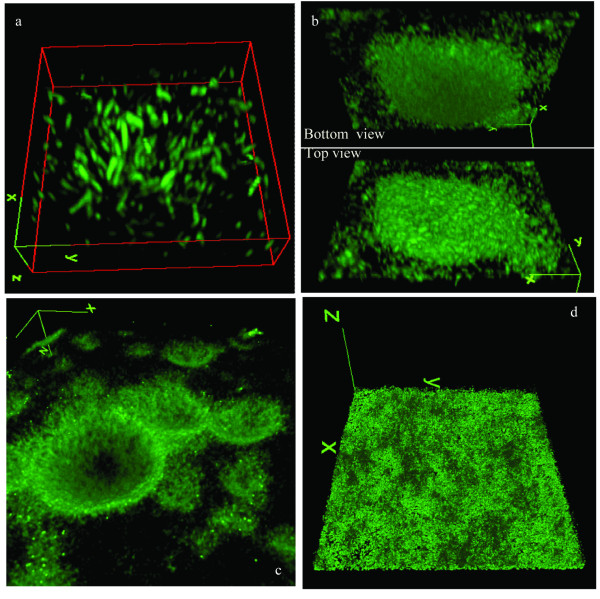
Three-dimensional reconstruction of *C. obsidiansis *biofilm structure formed on cellulose surface after a) 16 h, b) 24 h, c) 44 h and d) 68 h incubation.

**Figure 3:**
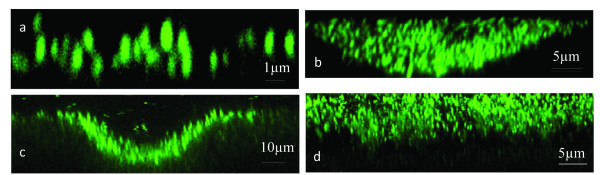
**Cross-sectional view of *C. obsidiansis *biofilm formed on a cellulose surface after a) 16 h, b) 24 h, c) 44 h and d) 68 h incubation**.

Initial attachment by *C. obsidiansis *to the cellulose substrate occurred during the first 16 h of incubation in the serum bottles. By 8 h after inoculation, single cells were observed randomly attaching to the cellulose surface (Figure 1b). These cells appeared to grow by cell division on the surface, forming small clusters of cells (Figure 1c). A three-dimensional reconstruction of one representative cluster is shown in Figure 2a. These data suggest that the cells are likely distributed as a monolayer on the cellulose surface. This observation is supported by a cross-sectional view of the cluster (Figure 3a). Interestingly, it appears that many of the cells are positioned vertically on the cellulose surface (Figures 2a and 3a). Whether this positioning is due to physical crowding of the cells on the surface or is the result of a specific attachment mechanism is the focus of ongoing studies.

### Inverted colony formation

By 24 h after inoculation, the formation of *C. obsidiansis *colonies was observed on the cellulose substrate (Figure 1d). The diameters of the colonies varied in size. Three-dimensional reconstructions of colony morphology revealed that these colonies were inverted; that is, the colonies were growing into the cellulose substrate rather than on the surface (Figure 2b). This inverted colony morphology can be seen clearly in the cross-sectional view (Figure 3b, Additional file 1). Measurements taken from this perspective indicate that the radius of the colony is larger than its height, with the width at 35 μm but the maximum depth at 10 μm. The formation of inverted colonies is likely due to cellulose hydrolysis by *C. obsidiansis*.

### Formation and fusion of crater-like depressions

As the experiment continued, the dimensions of the colonies continued to grow. By 44 h after inoculation, large depressions about 50 μm in width were observed on the cellulose substrate with adjacent depressions beginning to fuse (Figure 1e). Smaller depressions were also seen at this stage (Figure 1e). Three-dimensional reconstructions indicated depressions in the cellulose substrate were lined by *C. obsidiansis *cells (Figure 2c, Additional file 2). Measurements from a cross-sectional view indicate that the maximum biofilm thickness in the depression was about 10 μm (Figure 3c). By 48 h, multiple individual depressions had fused (Figure 1f) and by 56 h, the cellulose substrate was dominated by large, irregular (approximately 200 μm) depressions into the substratum (Figure 1g). From this point on, individual depressions could not be distinguished and the surface of the cellulose substrate was covered with a thin biofilm (Figure 1h). A three dimensional reconstruction of the cellulose substrate after 68 h incubation shows a rather uniform surface without any prominent cavities or depressions as seen in earlier time points (Figure 2d). The cross-sectional view shows that the biofilm thickness remains constant at approximately 10 μm after 68 h growth on the substrate (Figure 3d). At this point, it appears that a dynamic equilibrium was reached between biofilm growth and detachment, stabilizing the biofilm thickness at a constant value. Moreover, the cell density measured in this mature biofilm is about 1.69 × 10^11 ^cells cm^-3^, which is much greater than the cell density typically found in a biofilm grown on a soluble substrate (Zhang and Bishop 1994; Ito et al. 2002).

#### Cellulose hydrolysis

**Figure 4:**
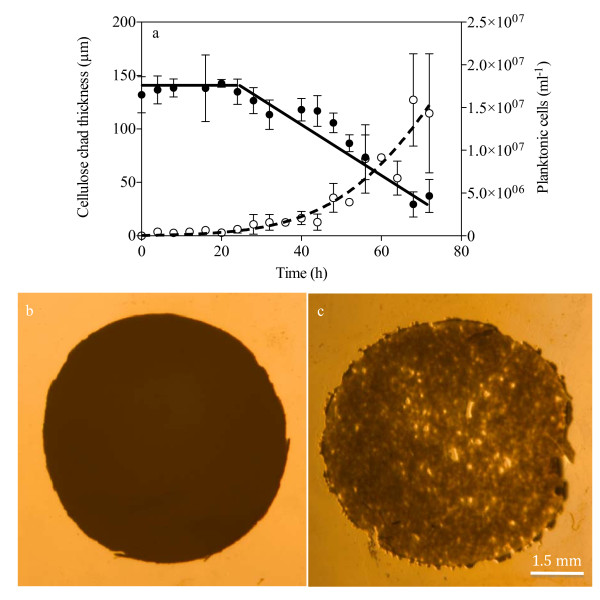
**Cellulose hydrolysis, a) reduction of cellulose chad thickness ('black circle') and measurement of planktonic cell concentration ('white circle') over time; and the cellulose chad morphology b) before and c) after 72 h incubation**.

It should be emphasized that the regenerated cellulose chad provides the sole carbon source for *C. obsidiansis *growth in this study. Hence, the hydrolysis of the cellulose chad occurs concurrently with biofilm formation. The change in chad thickness can be used as an indicator of cellulose hydrolysis and was measured throughout the experiment. The first measurable reduction in chad thickness was observed after 24 h incubation, which corresponds to the formation of inverted colonies (Figure 4a). From this point on, the cellulose chad thickness decreased at a nearly constant rate (Figure 4a). After 72 h incubation, the cellulose chad displayed significant degradation with irregular holes being visible (Figure 4c) in comparison with the new chad at the 0 h time point (Figure 4b). Our previous work indicated that a *C. obsidiansis *biofilm growing on cellulose generates more hydrolysate than it can utilize in order to establish an intra-biofilm substrate concentration high enough to support growth (Wang et al. 2011). The excess hydrolysate diffuses through the biofilm and is released into the bulk liquid where it can support planktonic cell growth (Wang et al. 2011).

#### Biofilm formation on linter cellulose

**Figure 5:**
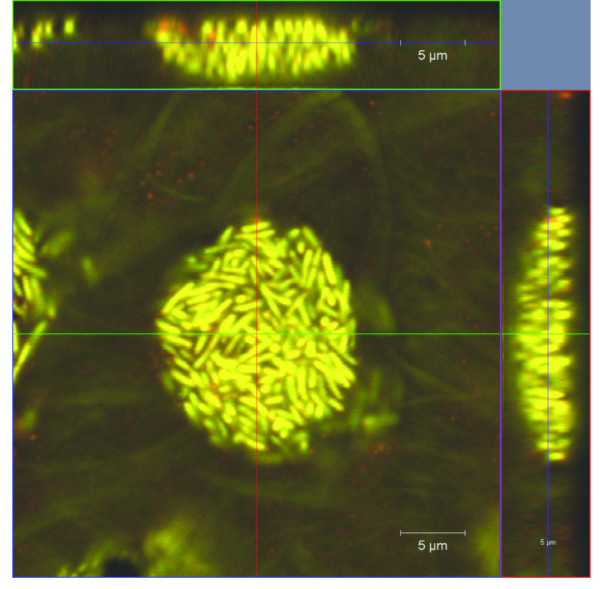
**Top and cross-sectional views of inverted colony formation by *C. obsidiansis *into the structure of linter cellulose chad after four days incubation**.

Although regenerated cellulose chads provide an ideal platform to image the process of biofilm formation and cellulose utilization (Figures 1, 2 and 3), it was unknown whether biofilm formation and degradation on natural cellulose occurred in the same manner. To address this question, a similar experiment was performed using linter cellulose, which is a natural cotton fiber containing higher crystallinity than regenerated cellulose (Gümüskaya et al. 2003). In order to create a flat surface for microscopy, linter cellulose chads were fabricated through a high-pressure homogenization method and used as the sole carbon source to culture *C. obsidiansis*. As with regenerated cellulose, biofilm growth on linter cellulose was characterized by the formation and fusion of depressions on the surface (Figure 5). *C. obsidiansis *biofilm formation on linter cellulose, however, was much slower than on regenerated cellulose, requiring four days to reach a growth stage comparable to 24 h growth on regenerated cellulose (compare Figure 3b with Figure 5). The higher crystallinity of linter cellulose likely accounts for this slower biofilm formation and cellulose degradation.

#### Biofilm formation by *C. thermocellum*

**Figure 6:**
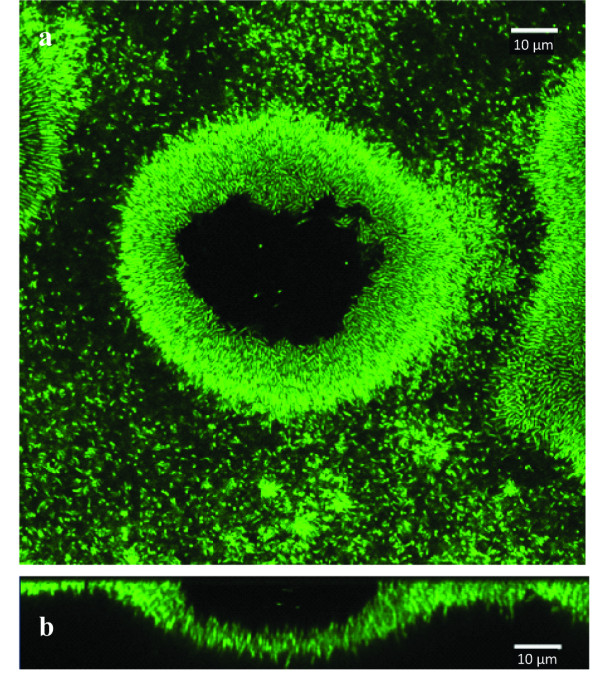
**Crater-like depression formed by *C. thermocellum *at 39 h.** a) top view, b) cross-sectional view.

In *C. thermocellum*, the cellulosome is thought to play important roles in promoting bacterial attachment to cellulose and in cellulose hydrolysis (Adams et al. 2006). *C. thermocellum *was used as a model cellulosome-producing organism to compare whether the presence of cellulosomes altered the dynamics of biofilm formation on cellulose compared to non-cellulosome producing bacteria. In this study, *C. thermocellum *was grown with regenerated cellulose chads as the sole carbon source. Results showed a very similar biofilm formation process to that of the *C. obsidiansis*, characterized by the formation of depressions in the cellulose substrate (Figure 6).

## Discussion

In this study, the spatial and temporal dynamics of biofilm formation by two different microorganisms on two different cellulose substrates were investigated and correlated to cellulose degradation. Previous studies of bacterial degradation of biomass in sheep rumen using electron microscopy showed the presence of bacteria within cavities on the plant wall, leading to the hypothesis that the cellulolytic bacteria used a tunneling mechanism to degrade the plant (Dinsdale et al. 1978). Similarly, after incubation with the ruminal cellulolytic bacteria *Ruminococcus flavefaciens*, cell-sized pits were observed on leaf sheaths which were presumed to be due to bacterial degradation (Shinkai and Kobayashi, 2007). In another study, Gehin et al. (1996) observed the attachment of *Clostridium cellulolyticum *on Whatman No. 1 filter paper after 30 minutes incubation, although colony formation was not observed during this short experiment.

**Figure 7:**
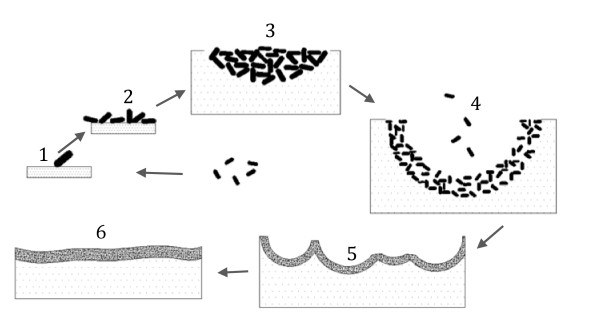
**Schematic illustration of the six stages of cellulolytic biofilm formation on cellulose surface observed from this study.** i.e., 1) single cell attachment to the substrate; 2) cell growth and division 3) inverted colony formation; 4) crater-like depression formation due to degradation of the cellulose substrate; 5) fusion of the depressions; and 6) homogenous biofilm formation.

The use of flat cellulose substrates coupled with sampling the biofilm structure at multiple stages of development allowed dissection of the multi-step process of biofilm formation and cellulose degradation (Figure 7). The process started with the random attachment of individual cells on the cellulose surface. These cells appear to grow and divide, forming colonies that grow into the substrate. The depressions formed by microbial hydrolysis of cellulose eventually fuse, resulting in a thin biofilm that covers the entire cellulose substrate. This biofilm formation and cellulose degradation process was observed not only on regenerated cellulose surface but also on natural linter cellulose surface (Figures 2 and 5). These data also confirm that cellulosomes are not required for the attachment of cellulolytic bacteria on cellulose surfaces, since the crater-like biofilm structure was observed for both cellulosome-producing and non-cellulosome producing cellulolytic bacteria (Figures 2 and 6). It is tempting to speculate that this colony development process represents a common cellulose degradation mechanism for cellulolytic bacteria, although additional bacteria and substrates should be tested.

**Figure 8:**
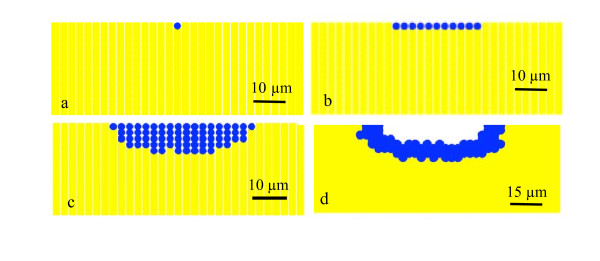
**Model of biofilm formation simulated by cellular automata.** a) initial bacteria attachment at 0 h; b) horizontal monolayer cluster development at 16 h, c) inverted colony formation at 24 h and d) crater-like depression formation at 44 h.

The key steps in cellulolytic biofilm formation were simulated with cellular automata. We used a "nine-neighbor square" model for a two-dimensional cellular automata in which both the nearest and next-nearest cells are considered. The cellulose substrate is represented by a 30 × 15 grid upon which a single cell is attached (Figure 8a), which is similar to the distribution of cells at the 8 h time point (Figure 1b). Using the doubling time reported for *C. obsidiansis *with Avicel as substrate (Hamilton-Brehm et al. 2009) and a horizontal division rule, a monolayer of cells is observed at 16 h (Figure 8b). Again, this distribution of cells is similar to the distribution observed experimentally (Figure 3a). By restricting the maximum biofilm thickness to the experimentally observed 10 μm through the cell detachment simulation and the application of both horizontal and vertical division rules, the model produced depressions in the cellulose surface (Figures 8c, d) that closely matched the dynamics of *C. obsidiansis *biofilm formation (Figures 3b, c). This simple simulation in Figure 8 further demonstrates the synchronized dynamics between biofilm formation and cellulose degradation. The reason why *C. obsidiansis *cells did not grow into the cellulose at 8 h and earlier might be attributable to the available peripheral substrate at the early stage. At later stages (16 h), the cells in the center of the colony have to move downward into the substrate in order to access carbon.

Judging from the correlation between *C. cellulyticum *activity and adhesion to cellulose, Lynd et al. (2006) predicted biofilm formation might facilitate cellulose degradation. The direct observation and measurement of biofilm formation and cellulose degradation in this study suggests that only the portions of the cellulose substrate colonized by the biofilm were effectively hydrolyzed. These data emphasize the critical role of biofilm formation in cellulose degradation. Hence, a rapid startup of cellulose hydrolysis is theoretically achievable by increasing the number of bacteria attached on the cellulose substrate during the initial phase until the maximum rate of hydrolysis is reached, correlating to complete substrate coverage by the biofilm. This saturation hydrolysis rate is about 5.33 × 10^-5 ^g h^-1 ^cm^-2 ^as measured from the linear degradation profile in Figure 4a. This kind of constant hydrolysis rate has been widely reported and thought to be the result of microbial attachment to all accessible substrate (Batstone et al. 2001). Consistent with this assumption, even a 3-fold increase in the number of planktonic cells did not increase the cellulose hydrolysis rate (Figure 4a), suggesting that cellulose hydrolysis is performed mainly by attached cells.

This study provides new information on the growth and structure of cellulolytic biofilms. After the initial attachment phase when the bacteria form inverted colonies and depressions in the substrate, the biofilm maintains a thin and uniform profile (approximately 10 μm) with a high cell concentration (between 10^11 ^to 10^12 ^cells cm^-3^) for the remainder of the experiment. These properties are in line with the cellulolytic biofilm morphologies analyzed in other studies, regardless of the type of feedstock or organism (Table 1). However, the cellulolytic biofilm morphology observed in this study as well as others appears quite different from the morphology of biofilms grown on soluble substrates which tend to display a heterogeneous structure with internal porosity (van Loosdrecht et al. 2002). Biofilms grown on soluble substrates typically display a thickness on the scale of 100 μm to 1000 μm and a cellular density under 10^11 ^cells cm^-3 ^(Zhang and Bishop 1994; Ito et al. 2002). It is worth mentioning that the biofilm thickness and cellular density are usually believed to be positively and negatively correlated with substrate availability, respectively (Park et al. 1998). High soluble substrate concentrations tend to promote growth of thick biofilms which are then subjected to mass diffusion limitations, leading to the formation of porous structures with fewer cells to facilitate substrate transfer (van Loosdrecht et al. 2002). Such a mass diffusion limitation results in an uneven growth rate within the soluble substrate feeding biofilm and leads to a heterogeneous biofilm morphology. In contrast, low soluble substrate availability supports only thin biofilms because mass diffusion is no longer a rate-limiting step, and thus dense and uniform biofilms are formed (Park et al. 1998). Our previous work on the modeling of hydrolysate diffusion and utilization in cellulose feeding biofilms are consistent with this inference (Wang et al. 2011). These modeling studies predicted that the hydrolysate concentration profile is quite uniform throughout the cellulolytic biofilm and that the growth of the biofilm is limited by hydrolysate utilization rates, rather than hydrolysate diffusion rates (Wang et al. 2011).

**Table 1:** Thickness and cell density of cellulolytic biofilms cultivated with various types of feedstock and microorganisms

**No**.	Substrate	Culture	Thickness	**ρ**_**a **_**(cells cm**^**-2**^**)**	d (μm)	Microscope	**ρ**_**v **_**(cells cm**^**-3**^**)**	Reference
1	Alfalfa leave	Mixed rumen bacteria	Monolayer	2.12 × 10^8^	0.77	TEM	2.74 × 10^12^	(Cheng et al. 1984)
2	Forage	*Fibrobacter succinogenes*	Monolayer	9.68 × 10^7^	1.15	SEM	8.43 × 10^11^	(Weimer et al. 1993)
3	Wheat straw	*Fibrobacter succinogenes Butyrivibrio fibrisolvens*	Monolayer	6.85 × 10^7^	1.36	SEM	5.02 × 10^11^	(Miron et al. 2001)
4	Cellulose	Land fill mixed culture	Monolayer	2.02 × 10^7^	2.51	Confocal	8.05 × 10^10^	(Burrell et al. 2004)
5	Wheat embryo	*Agrobacterium tumefaciens*	Monolayer	5.29 × 10^7^	1.55	SEM	3.41 × 10^11^	(Mooney and Goodwin 1991)
6	Cellulose	Mixed leachate	Monolayer	2.53 × 10^7^	2.25	SEM	1.13 × 10^11^	(Song et al. 2005)
7	Cellulose	*C. obsidiansis*	~ 10 μm	1.69 × 10^8^	1.80	Confocal	1.69 × 10^11^	This study

## Competing interests

The authors declare that they have no competing interests.

## Supplementary Material

Additional file 1 ***C. obsidiansis *biofilm formation at 24 h**. Visualization of the three-dimensional structure of an inverted colony of *C. obsidiansis *growing into regenerated cellulose substrate at 24 h Click here for file

Additional file 2 ***C. obsidiansis *biofilm formation at 44 h**. Visualization showing the three-dimensional structure of crater-like depressions formed by *C. obsidiansis *on regenerated cellulose at 44 h Click here for file
